# Error in Figure

**DOI:** 10.1001/jamanetworkopen.2026.18475

**Published:** 2026-05-26

In the Original Article titled “Clinical Outcomes in Bacillus Calmette-Guérin–Exposed Non–Muscle-Invasive Bladder Cancer,”^1^ published April 16, 2026, the number of patients exposed to Bacillus Calmette-Guérin therapy should have been 2604 rather than 2605. This article has been corrected.^1^
